# *catena*-Poly[[(2,2′-bipyridine-κ^2^ *N*,*N*′)cadmium]-μ_3_-4-nitro­phthalato-κ^4^ *O*:*O*′,*O*′′:*O*′′′]

**DOI:** 10.1107/S1600536811000468

**Published:** 2011-01-15

**Authors:** Yang Fan, Guo-Min Xu, Hai-Ting Lu, Wei Li

**Affiliations:** a College of Chemistry and Chemical Engineering, Xinyang Normal University, Xinyang 464000, People’s Republic of China; b National Engineering Research Center for Compounding and Modification of Polymeric Materials, Guiyang 550014, People’s Republic of China

## Abstract

In the title polymeric compound, [Cd(C_8_H_3_NO_6_)(C_10_H_8_N_2_)]_*n*_, two O atoms from both carboxyl­ate groups of a nitro­phthalate anion coordinate to the Cd^II^ cation, forming a seven-membered chelate ring and two carboxyl­ate O atoms from another two nitro­phthalate anions and a 2,2′-bipyridine ligand coordinate to the Cd cation to complete the distorted octa­hedral coordination geometry. The carboxyl­ate groups of the nitro­phthalate anion adopt a *syn–anti* bridging mode, linking adjacent Cd^II^ cations and forming a polymeric chain running along the *a* axis. Weak intra- and inter­molecular C—H⋯O hydrogen bonding is present in the crystal structure.

## Related literature

For applications of coordination polymers, see: Long & Yaghi (2009 ▶); Kurmoo *et al.* (2009 ▶); Cheetham *et al.* (2006 ▶). For related complexes with 4-nitro­phthalate ligands, see: Guo & Guo (2007 ▶); Xu *et al.* (2009 ▶); He *et al.* (2010 ▶).
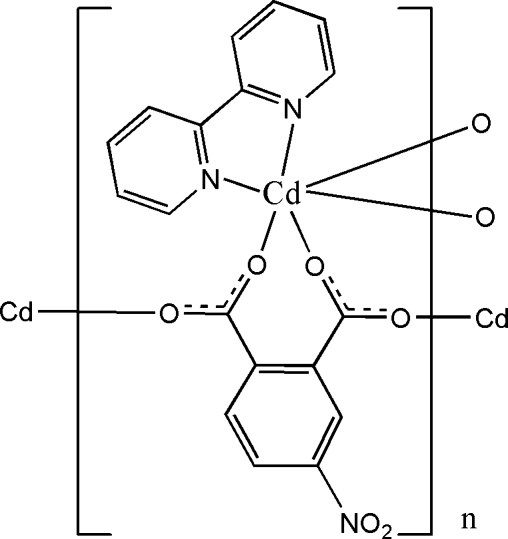


## Experimental

### 

#### Crystal data

[Cd(C_8_H_3_NO_6_)(C_10_H_8_N_2_)]
*M* *_r_* = 477.70
Monoclinic, 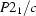

*a* = 7.3327 (4) Å
*b* = 17.3786 (9) Å
*c* = 13.3859 (7) Å
β = 98.149 (2)°
*V* = 1688.57 (15) Å^3^
*Z* = 4
Mo *K*α radiation
μ = 1.34 mm^−1^
*T* = 293 K
0.50 × 0.30 × 0.07 mm

#### Data collection

Bruker APEXII CCD area-detector diffractometer
Absorption correction: multi-scan (*SADABS*; Sheldrick, 1996 ▶) *T*_min_ = 0.624, *T*_max_ = 0.911
19676 measured reflections
3825 independent reflections
3452 reflections with *I* > 2σ(*I*)
*R*_int_ = 0.027

#### Refinement

*R*[*F* ^2^ > 2σ(*F* ^2^)] = 0.022
*wR*(*F* ^2^) = 0.053
*S* = 1.03
3825 reflections
253 parameters
H-atom parameters constrained
Δρ_max_ = 0.44 e Å^−3^
Δρ_min_ = −0.31 e Å^−3^

### 

Data collection: *APEX2* (Bruker, 2007 ▶); cell refinement: *SAINT* (Bruker, 2007 ▶); data reduction: *SAINT*; program(s) used to solve structure: *SHELXTL* (Sheldrick, 2008 ▶); program(s) used to refine structure: *SHELXTL*; molecular graphics: *SHELXTL*; software used to prepare material for publication: *SHELXTL*.

## Supplementary Material

Crystal structure: contains datablocks I, global. DOI: 10.1107/S1600536811000468/xu5128sup1.cif

Structure factors: contains datablocks I. DOI: 10.1107/S1600536811000468/xu5128Isup2.hkl

Additional supplementary materials:  crystallographic information; 3D view; checkCIF report

## Figures and Tables

**Table 1:** Selected bond lengths (Å)

Cd1—O1^i^	2.2820 (15)
Cd1—O2	2.3165 (14)
Cd1—O3^ii^	2.3570 (15)
Cd1—O4	2.4753 (16)
Cd1—N2	2.3659 (18)
Cd1—N3	2.3979 (17)

Symmetry codes: (i) 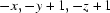; (ii) 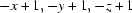.

**Table 2:** Hydrogen-bond geometry (Å, °)

*D*—H⋯*A*	*D*—H	H⋯*A*	*D*⋯*A*	*D*—H⋯*A*
C5—H5⋯O5^iii^	0.93	2.49	3.349 (3)	154
C9—H9⋯O3^ii^	0.93	2.39	3.037 (3)	126
C12—H12⋯O3^iv^	0.93	2.56	3.490 (3)	177
C15—H15⋯O3^iv^	0.93	2.56	3.493 (3)	176
C18—H18⋯O2^i^	0.93	2.43	3.235 (3)	145

Symmetry codes: (i) 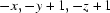; (ii) 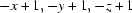; (iii) 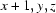; (iv) 
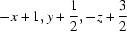
.

**Fig. 1.**
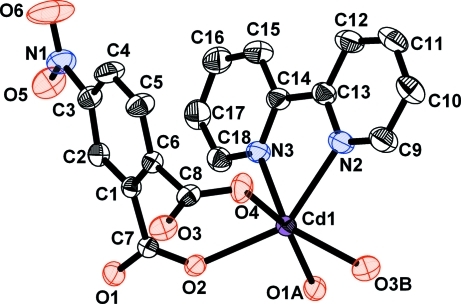
The molecular structure of the title compound with the atom-numbering scheme. Displacement ellipsoids are drawn at the 50% probability level. Hydrogen atoms are omitted for clarity. [Symmetry code: (A) -x, 1 - y, 1 - z; (B) 1 - x, 1 - y, 1 - z.]

**Fig. 2.**
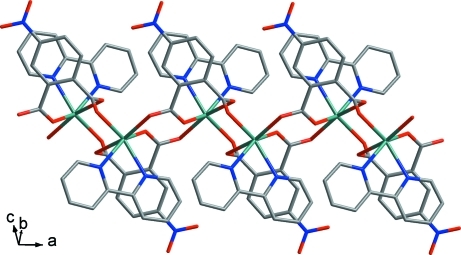
The one-dimensional structure of the title compound. Hydrogen atoms are omitted for clarity.

## supplementary crystallographic information

### Comment

The rational design and synthesis of coordination complexes and polymers have attracted considerable attention since they can exhibit various fascinating structure topologies and have potential applications in gas adsorption and magnetism (Long & Yaghi, 2009; Kurmoo *et al.*, 2009). During the past decades, large amount of coordination complexes and polymers have been successfully prepared and reported, in which polycarboxylates have been widely used as bridging ligands to construct coordination complexes and polymers (Cheetham *et al.*, 2006). 4-Nitrophthalic acid is a good candidate in the polycarboxylate family because it has two carboxylate groups that can supply four potential O-donor atoms. However, only a few reports exist of coordination complexes and polymers related to 4-nitrophthalic acid have been published to our knowledge (Guo *et al.*, 2007; Xu *et al.*, 2009; He *et al.*, 2010). In order to enrich the metal-4-nitrophthalate coordination complexes and polymers, we employed this ligand to assemble with cadmium ion in the presence of ancillary 2,2'-bipyridine ligand and obtained the title one-dimensional coordination polymer [Cd(4-nitrophthalate)(2,2'-bpy)]_n_.

As shown from Fig. 1, the asymmetric unit of the title compound (I) has a Cd(II) ion, a 4-nitrophthalate and a 2,2'-bipyridine ligand. Cd1 ion has a distorted octahedral coordination environment comprising of two nitrogen atoms from a chelating 2,2'-bipyridine ligand, two oxygen atoms from both of the *syn-anti* carboxylates of a chelating 4-nitrophthalate ligand and two oxygen atoms from other two *syn-anti* carboxylates of two different crystallographic symmetric 4-nitrophthalate ligands. Each Cd(II) ion is linked to adjacent two Cd(II) ions by two *syn-anti* carboxylates from one 4-nitrophthalate ligand and other two *syn–anti* carboxylates from two different 4-nitrophthalate ligands to form a chained structure along the *a* axis with alternating Cd···Cd distances of 4.198 (5) and 5.094 (1)Å (Fig. 2).

### Experimental

Cd(NO_3_)_2_.4H_2_O (0.25 mmol, 0.077 g), 4-nitrophthalic acid (0.25 mmol, 0.052 g), 2,2'-bipyridine (0.25 mmol, 0.039 g) and NaOH (0.5 mmol, 0.020 g) were well mixed in 8 ml distilled water, and the solution was stirred for 30 min and then transferred into a 23 ml Teflon-lined bomb at 423 K for 3 d and slowly cooled to room temperature. Colorless crystals suitable for X-ray analysis were obtained.

### Refinement

H atoms were placed in calculated positions with C—H = 0.93 Å and refined in riding mode, U_iso_(H) = 1.2U_eq_(C).

### Crystal data

**Table:**

[Cd(C_8_H_3_NO_6_)(C_10_H_8_N_2_)]	*F*(000) = 944
*M**_r_* = 477.70	*D*_x_ = 1.879 Mg m^−^^3^
Monoclinic, *P*2_1_/*c*	Mo *K*α radiation, λ = 0.71073 Å
Hall symbol: -P 2ybc	Cell parameters from 5075 reflections
*a* = 7.3327 (4) Å	θ = 2.8–27.6°
*b* = 17.3786 (9) Å	µ = 1.34 mm^−^^1^
*c* = 13.3859 (7) Å	*T* = 293 K
β = 98.149 (2)°	Sheet, colorless
*V* = 1688.57 (15) Å^3^	0.50 × 0.30 × 0.07 mm
*Z* = 4	

### Data collection

**Table:**

Bruker APEXII CCD area-detector diffractometer	3825 independent reflections
Radiation source: fine-focus sealed tube	3452 reflections with *I* > 2σ(*I*)
graphite	*R*_int_ = 0.027
φ and ω scans	θ_max_ = 27.5°, θ_min_ = 1.9°
Absorption correction: multi-scan (*SADABS*; Sheldrick, 1996)	*h* = −9→9
*T*_min_ = 0.624, *T*_max_ = 0.911	*k* = −21→22
19676 measured reflections	*l* = −17→17

### Refinement

**Table:**

Refinement on *F*^2^	Primary atom site location: structure-invariant direct methods
Least-squares matrix: full	Secondary atom site location: difference Fourier map
*R*[*F*^2^ > 2σ(*F*^2^)] = 0.022	Hydrogen site location: inferred from neighbouring sites
*wR*(*F*^2^) = 0.053	H-atom parameters constrained
*S* = 1.03	*w* = 1/[σ^2^(*F*_o_^2^) + (0.0234*P*)^2^ + 1.1148*P*] where *P* = (*F*_o_^2^ + 2*F*_c_^2^)/3
3825 reflections	(Δ/σ)_max_ = 0.003
253 parameters	Δρ_max_ = 0.44 e Å^−^^3^
0 restraints	Δρ_min_ = −0.31 e Å^−^^3^

### Special details

**Table:**

Experimental. Calcd for C_18_H_11_N_3_O_6_Cd (Mr = 477.71): C, 45.26; H, 2.32; N, 8.80%. Found: C, 45.34; H, 2.27; N, 8.85%. FT—IR (KBr) 3450 b, 3099 w, 3068 w, 3037 w, 1590 vs, 1551 m, 1513 s, 1495 s, 1439 s, 1422 s,1392 s, 1360 s, 1316 w, 1245 m, 1170 m, 1161 m, 1066 w, 1016 s, 905 m, 830 s,771 s, 740 s, 725 w. Thermogravimetric analysis (TGA) shows that compound (I) has a good thermal stability and exhibits no weight loss untill 200 °C.
Geometry. All e.s.d.'s (except the e.s.d. in the dihedral angle between two l.s. planes) are estimated using the full covariance matrix. The cell e.s.d.'s are taken into account individually in the estimation of e.s.d.'s in distances, angles and torsion angles; correlations between e.s.d.'s in cell parameters are only used when they are defined by crystal symmetry. An approximate (isotropic) treatment of cell e.s.d.'s is used for estimating e.s.d.'s involving l.s. planes.
Refinement. Refinement of *F*^2^ against ALL reflections. The weighted *R*-factor *wR* and goodness of fit *S* are based on *F*^2^, conventional *R*-factors *R* are based on *F*, with *F* set to zero for negative *F*^2^. The threshold expression of *F*^2^ > σ(*F*^2^) is used only for calculating *R*-factors(gt) *etc*. and is not relevant to the choice of reflections for refinement. *R*-factors based on *F*^2^ are statistically about twice as large as those based on *F*, and *R*- factors based on ALL data will be even larger.

### Fractional atomic coordinates and isotropic or equivalent isotropic displacement parameters (Å^2^)

**Table:**

	*x*	*y*	*z*	*U*_iso_*/*U*_eq_	
Cd1	0.285437 (18)	0.580935 (8)	0.487418 (10)	0.02454 (6)	
C10	0.7094 (3)	0.77702 (15)	0.51077 (19)	0.0466 (6)	
H10	0.8100	0.7885	0.4782	0.056*	
O1	−0.0364 (2)	0.41748 (8)	0.63597 (11)	0.0330 (3)	
O3	0.5668 (2)	0.41313 (9)	0.65772 (12)	0.0361 (3)	
O2	0.17615 (18)	0.46901 (8)	0.55187 (10)	0.0279 (3)	
O4	0.5555 (2)	0.53373 (10)	0.60322 (11)	0.0400 (4)	
C6	0.3977 (3)	0.50255 (11)	0.74051 (14)	0.0250 (4)	
N3	0.1434 (2)	0.65808 (10)	0.60339 (13)	0.0311 (4)	
C7	0.1086 (2)	0.45463 (11)	0.63060 (14)	0.0233 (4)	
N1	0.0788 (3)	0.57744 (11)	0.96042 (15)	0.0440 (5)	
C1	0.2082 (2)	0.48689 (11)	0.72851 (13)	0.0233 (4)	
N2	0.4489 (3)	0.69724 (10)	0.52417 (14)	0.0365 (4)	
C2	0.1054 (3)	0.50831 (11)	0.80376 (14)	0.0269 (4)	
H2	−0.0194	0.4966	0.7979	0.032*	
C3	0.1915 (3)	0.54717 (13)	0.88696 (15)	0.0327 (4)	
C14	0.2403 (3)	0.71869 (12)	0.64530 (15)	0.0327 (4)	
C4	0.3784 (3)	0.56177 (15)	0.90138 (17)	0.0415 (5)	
H4	0.4336	0.5869	0.9593	0.050*	
C5	0.4821 (3)	0.53847 (14)	0.82832 (16)	0.0373 (5)	
H5	0.6086	0.5468	0.8377	0.045*	
C16	0.0198 (4)	0.73882 (15)	0.75846 (19)	0.0498 (6)	
H16	−0.0202	0.7655	0.8115	0.060*	
C17	−0.0804 (3)	0.67790 (14)	0.71452 (18)	0.0434 (5)	
H17	−0.1898	0.6630	0.7365	0.052*	
C18	−0.0145 (3)	0.63920 (12)	0.63669 (16)	0.0348 (5)	
H18	−0.0828	0.5983	0.6062	0.042*	
C15	0.1801 (4)	0.76022 (14)	0.72341 (18)	0.0460 (6)	
H15	0.2477	0.8021	0.7517	0.055*	
O5	−0.0883 (3)	0.57084 (11)	0.94263 (14)	0.0521 (5)	
C8	0.5143 (2)	0.48150 (12)	0.66069 (14)	0.0273 (4)	
C13	0.4092 (3)	0.74003 (12)	0.60168 (16)	0.0331 (4)	
O6	0.1566 (3)	0.60768 (16)	1.03689 (18)	0.0875 (8)	
C12	0.5195 (3)	0.80256 (13)	0.63640 (18)	0.0418 (5)	
H12	0.4917	0.8317	0.6905	0.050*	
C9	0.5963 (3)	0.71551 (14)	0.48055 (19)	0.0444 (6)	
H9	0.6238	0.6853	0.4273	0.053*	
C11	0.6697 (3)	0.82089 (14)	0.5902 (2)	0.0462 (6)	
H11	0.7437	0.8627	0.6125	0.055*	

### Atomic displacement parameters (Å^2^)

**Table:**

	*U*^11^	*U*^22^	*U*^33^	*U*^12^	*U*^13^	*U*^23^
Cd1	0.02554 (9)	0.02418 (9)	0.02423 (8)	−0.00343 (5)	0.00470 (6)	−0.00128 (5)
C10	0.0415 (14)	0.0370 (13)	0.0619 (16)	−0.0108 (10)	0.0092 (12)	−0.0012 (11)
O1	0.0276 (7)	0.0398 (9)	0.0307 (7)	−0.0110 (6)	0.0014 (6)	0.0029 (6)
O3	0.0315 (8)	0.0406 (9)	0.0370 (8)	0.0067 (6)	0.0079 (6)	−0.0058 (6)
O2	0.0283 (7)	0.0326 (8)	0.0230 (7)	−0.0045 (6)	0.0045 (5)	−0.0015 (5)
O4	0.0322 (8)	0.0532 (10)	0.0360 (8)	−0.0075 (7)	0.0097 (6)	0.0086 (7)
C6	0.0229 (9)	0.0276 (10)	0.0245 (9)	0.0005 (7)	0.0035 (7)	0.0007 (7)
N3	0.0359 (10)	0.0253 (9)	0.0314 (9)	0.0009 (7)	0.0026 (7)	−0.0025 (7)
C7	0.0216 (9)	0.0226 (9)	0.0253 (9)	0.0017 (7)	0.0015 (7)	0.0004 (7)
N1	0.0506 (13)	0.0439 (12)	0.0410 (11)	−0.0024 (9)	0.0183 (10)	−0.0138 (9)
C1	0.0239 (9)	0.0229 (9)	0.0232 (9)	0.0011 (7)	0.0033 (7)	0.0014 (7)
N2	0.0397 (10)	0.0297 (9)	0.0403 (10)	−0.0058 (8)	0.0065 (8)	−0.0051 (8)
C2	0.0232 (9)	0.0292 (10)	0.0290 (10)	0.0019 (8)	0.0065 (8)	0.0019 (8)
C3	0.0374 (11)	0.0334 (11)	0.0292 (10)	0.0018 (9)	0.0112 (9)	−0.0046 (8)
C14	0.0424 (12)	0.0250 (10)	0.0298 (10)	0.0021 (9)	0.0016 (9)	−0.0016 (8)
C4	0.0394 (13)	0.0540 (14)	0.0305 (11)	−0.0083 (11)	0.0029 (9)	−0.0149 (10)
C5	0.0257 (10)	0.0533 (14)	0.0325 (11)	−0.0066 (9)	0.0022 (9)	−0.0087 (10)
C16	0.0688 (17)	0.0414 (14)	0.0428 (13)	0.0051 (12)	0.0198 (12)	−0.0086 (11)
C17	0.0475 (14)	0.0390 (13)	0.0461 (13)	0.0065 (10)	0.0157 (11)	0.0033 (10)
C18	0.0379 (12)	0.0280 (11)	0.0385 (11)	0.0023 (9)	0.0051 (9)	0.0007 (9)
C15	0.0609 (16)	0.0359 (13)	0.0414 (13)	−0.0025 (11)	0.0075 (11)	−0.0114 (10)
O5	0.0425 (10)	0.0660 (12)	0.0510 (10)	0.0134 (8)	0.0179 (8)	−0.0088 (9)
C8	0.0174 (9)	0.0397 (12)	0.0243 (9)	−0.0034 (8)	0.0013 (7)	−0.0032 (8)
C13	0.0388 (12)	0.0249 (10)	0.0337 (10)	0.0007 (9)	−0.0011 (9)	0.0001 (8)
O6	0.0757 (15)	0.121 (2)	0.0728 (15)	−0.0328 (15)	0.0343 (12)	−0.0668 (15)
C12	0.0493 (14)	0.0288 (12)	0.0448 (13)	−0.0029 (10)	−0.0016 (11)	−0.0065 (9)
C9	0.0452 (14)	0.0385 (13)	0.0514 (14)	−0.0088 (10)	0.0130 (11)	−0.0079 (11)
C11	0.0466 (14)	0.0287 (12)	0.0603 (15)	−0.0109 (10)	−0.0030 (12)	−0.0031 (11)

### Geometric parameters (Å, °)

**Table:**

Cd1—O1^i^	2.2820 (15)	N2—C9	1.337 (3)
Cd1—O2	2.3165 (14)	N2—C13	1.342 (3)
Cd1—O3^ii^	2.3570 (15)	C2—C3	1.377 (3)
Cd1—O4	2.4753 (16)	C2—H2	0.9300
Cd1—N2	2.3659 (18)	C3—C4	1.380 (3)
Cd1—N3	2.3979 (17)	C14—C15	1.393 (3)
C10—C11	1.372 (4)	C14—C13	1.489 (3)
C10—C9	1.378 (3)	C4—C5	1.382 (3)
C10—H10	0.9300	C4—H4	0.9300
O1—C7	1.255 (2)	C5—H5	0.9300
O3—C8	1.251 (2)	C16—C17	1.373 (4)
O2—C7	1.252 (2)	C16—C15	1.377 (4)
O4—C8	1.254 (2)	C16—H16	0.9300
C6—C5	1.396 (3)	C17—C18	1.384 (3)
C6—C1	1.403 (3)	C17—H17	0.9300
C6—C8	1.505 (3)	C18—H18	0.9300
N3—C18	1.339 (3)	C15—H15	0.9300
N3—C14	1.347 (3)	C13—C12	1.395 (3)
C7—C1	1.515 (3)	C12—C11	1.375 (4)
N1—O6	1.218 (3)	C12—H12	0.9300
N1—O5	1.220 (3)	C9—H9	0.9300
N1—C3	1.469 (3)	C11—H11	0.9300
C1—C2	1.392 (3)		
			
O1^i^—Cd1—O2	89.78 (5)	C3—C2—H2	120.6
O1^i^—Cd1—O3^ii^	79.46 (5)	C1—C2—H2	120.6
O2—Cd1—O3^ii^	124.57 (5)	C2—C3—C4	122.41 (18)
O1^i^—Cd1—N2	118.03 (6)	C2—C3—N1	118.68 (19)
O2—Cd1—N2	146.28 (6)	C4—C3—N1	118.84 (19)
O3^ii^—Cd1—N2	81.69 (6)	N3—C14—C15	120.9 (2)
O1^i^—Cd1—N3	94.91 (6)	N3—C14—C13	116.74 (18)
O2—Cd1—N3	91.34 (5)	C15—C14—C13	122.3 (2)
O3^ii^—Cd1—N3	143.29 (6)	C3—C4—C5	118.8 (2)
N2—Cd1—N3	68.99 (6)	C3—C4—H4	120.6
O1^i^—Cd1—O4	160.78 (5)	C5—C4—H4	120.6
O2—Cd1—O4	77.10 (5)	C4—C5—C6	120.3 (2)
O3^ii^—Cd1—O4	96.33 (5)	C4—C5—H5	119.8
N2—Cd1—O4	79.41 (6)	C6—C5—H5	119.8
N3—Cd1—O4	99.33 (6)	C17—C16—C15	119.6 (2)
C11—C10—C9	118.3 (2)	C17—C16—H16	120.2
C11—C10—H10	120.9	C15—C16—H16	120.2
C9—C10—H10	120.9	C16—C17—C18	118.3 (2)
C7—O1—Cd1^i^	123.37 (12)	C16—C17—H17	120.8
C8—O3—Cd1^ii^	99.45 (12)	C18—C17—H17	120.8
C7—O2—Cd1	132.87 (12)	N3—C18—C17	122.8 (2)
C8—O4—Cd1	112.52 (12)	N3—C18—H18	118.6
C5—C6—C1	119.77 (17)	C17—C18—H18	118.6
C5—C6—C8	118.59 (17)	C16—C15—C14	119.5 (2)
C1—C6—C8	121.64 (17)	C16—C15—H15	120.3
C18—N3—C14	118.85 (18)	C14—C15—H15	120.3
C18—N3—Cd1	123.62 (14)	O3—C8—O4	124.44 (18)
C14—N3—Cd1	117.07 (14)	O3—C8—C6	117.51 (17)
O2—C7—O1	126.20 (17)	O4—C8—C6	118.04 (18)
O2—C7—C1	117.06 (16)	N2—C13—C12	120.7 (2)
O1—C7—C1	116.71 (16)	N2—C13—C14	116.68 (19)
O6—N1—O5	122.8 (2)	C12—C13—C14	122.6 (2)
O6—N1—C3	118.4 (2)	C11—C12—C13	119.6 (2)
O5—N1—C3	118.72 (19)	C11—C12—H12	120.2
C2—C1—C6	119.66 (17)	C13—C12—H12	120.2
C2—C1—C7	118.75 (16)	N2—C9—C10	123.1 (2)
C6—C1—C7	121.28 (16)	N2—C9—H9	118.5
C9—N2—C13	118.92 (19)	C10—C9—H9	118.5
C9—N2—Cd1	121.94 (15)	C10—C11—C12	119.4 (2)
C13—N2—Cd1	118.37 (14)	C10—C11—H11	120.3
C3—C2—C1	118.88 (18)	C12—C11—H11	120.3

Symmetry codes: (i) −*x*, −*y*+1, −*z*+1; (ii) −*x*+1, −*y*+1, −*z*+1.

### Hydrogen-bond geometry (Å, °)

**Table:**

*D*—H···*A*	*D*—H	H···*A*	*D*···*A*	*D*—H···*A*
C5—H5···O5^iii^	0.93	2.49	3.349 (3)	154
C9—H9···O3^ii^	0.93	2.39	3.037 (3)	126
C12—H12···O3^iv^	0.93	2.56	3.490 (3)	177
C15—H15···O3^iv^	0.93	2.56	3.493 (3)	176
C18—H18···O2^i^	0.93	2.43	3.235 (3)	145

Symmetry codes: (iii) *x*+1, *y*, *z*; (ii) −*x*+1, −*y*+1, −*z*+1; (iv) −*x*+1, *y*+1/2, −*z*+3/2; (i) −*x*, −*y*+1, −*z*+1.
